# Propofol-Induced Mitochondrial Dysfunction Is Independent of Mitochondrial Permeability Transition

**DOI:** 10.3390/biomedicines13123125

**Published:** 2025-12-18

**Authors:** Aya Kawachi, Shoichiro Shibata, Eskil Elmér, Hiroyuki Uchino

**Affiliations:** 1 Department of Anesthesiology, Tokyo Medical University, Tokyo 1600023, Japan; 2 Mitochondrial Medicine, Department of Clinical Sciences, Lund University, 22184 Lund, Sweden

**Keywords:** propofol, mitochondrial respiratory capacity, mitochondrial swelling, mitochondrial dysfunction, mitochondrial permeability transition pore, cyclophilin D

## Abstract

**Background/Objectives**: In recent years, it has been suggested that sedatives may cause brain damage. One possible mechanism is interference with oxidative phosphorylation of brain mitochondria, but much remains unknown. In this study, we focused on dexmedetomidine, midazolam, and propofol, essential sedatives in anesthesia and intensive care, and aimed to understand the effects of these drugs on mouse brain mitochondria. **Methods**: We measured changes in mitochondrial respiratory capacity and swelling rate upon exposure to these sedatives in a wide concentration range. For the sedative that demonstrated impaired mitochondrial function we explored the possible involvement of mitochondrial permeability transition pore opening using brain mitochondria from cyclophilin D knockout (CypD KO) mice and detected cytochrome c (cyt c) release by Western blot. **Results**: Of the three sedatives, only high concentrations of propofol exhibited reduced respiratory capacity and mitochondrial swelling, toxicity which was not prevented by CypD KO. Furthermore, propofol did not induce cyt c release. **Conclusions:** These results suggest that propofol-induced brain mitochondrial dysfunction is a mechanism independent of mPTP opening.

## 1. Introduction

Many drugs are used in the fields of anesthesia and intensive care. Among them, various drugs are known to have a neuroprotective effect, including the local anesthetic lidocaine, β-receptor blockers, and the NMDA receptor antagonist ketamine [1,2,3]. Other intravenous anesthetics that have been shown to have cerebral protective effects include the alpha receptor antagonist dexmedetomidine, the GABA_A_ receptor antagonist propofol (2,6-diisopropylphenol), and the benzodiazepine midazolam [4,5,6]. Compared to other general anesthetics, propofol has advantages such as a fast onset of action, a short half-life, a fast recovery after discontinuation of administration, and few side effects, making it a type of sedative commonly used for the induction and maintenance of general anesthesia. However, in recent years, it has been proposed that intravenous anesthetics not only have a neuroprotective effect but also have the potential to cause brain damage [7,8]. In particular, propofol is known to cause propofol infusion syndrome (PRIS) by long-term treatment with high doses, inhibition of mitochondrial respiratory function has been suggested as one of the mechanisms of propofol-induced brain damage [9,10,11], and some reports have suggested that this is due to energetic failure caused by the opening of the mitochondrial permeability transition pore (mPTP) and the swelling-induced release of apoptosis-inducing factors such as cytochrome c (cyt c) [12,13]. The mPTP is a nonselective channel complex formed at the junction between the inner and outer mitochondrial membranes. It is involved in normal physiological functions such as cellular energy production and calcium ion homeostasis. On the other hand, under certain conditions such as an increase in calcium ion concentration, it can trigger apoptosis by opening its pore to release cell death-inducing factors such as cyt c and activating caspase 9 [14]. Mitochondria in nerve cells are particularly vulnerable due to their high energy demands and susceptibility to reactive oxygen species produced during the energy production process. Research into brain mitochondria will contribute greatly to elucidating and treating brain damage caused by neurodegenerative diseases and other conditions. However, despite accumulating evidence that several sedatives can inhibit mitochondrial respiration or cause mPTP opening, it remains unclear whether this mechanism is shared among commonly used intravenous sedatives with different receptor systems, such as dexmedetomidine, midazolam, and propofol. Therefore, in this study, we aimed to investigate the effects of dexmedetomidine, midazolam, and propofol, which are representative anesthetics with distinct receptor systems and intensive care drugs, on mitochondrial respiratory function, swelling rate, and mPTP opening, in order to gain more detailed insight into mitochondrial dysfunction caused by intravenous anesthetics. This study also compared the mice with those lacking the mPTP component cyclophilin D (CypD) gene. It has been suggested that inhibition of CypD improves pathologies associated with mitochondrial dysfunction [9,15], such as Parkinson’s disease [16], Alzheimer’s disease [17], amyotrophic lateral sclerosis [18], and Huntington’s disease [19]. It was therefore hypothesized that if the mechanism of mitochondrial dysfunction caused by sedatives is mediated by mPTP, it could be prevented by CypD knockout (KO).

## 2. Materials and Methods

### 2.1. Animals

The design of this study was approved by the Animal Care and Use Committee of Tokyo Medical University (study approval number: R3-0037), and all animal procedures were carried out in accordance with institutional and national guidelines for animal experiments. Male wild-type (WT) C57BL/6J mice (10–12 weeks old, weight 25–30 g) were purchased from CLEA Japan, Inc. (Tokyo, Japan). Male whole-body CypD KO (Ppif^−/−^) mice were generated from heterozygous breeding pairs derived from a male KO mouse (#022308 B6;129-Ppiftm1 Jmol/J; The Jackson Laboratory, Bar Harbor, ME, USA) and a female WT mouse (C57BL/6J; CLEA Japan, Inc.). Total of 64 WT mice (25 mice for mitochondrial respiratory capacity assay, 24 mice for mitochondrial swelling assay and 15 mice Western blot (WB) analysis, respectively) and 17 CypD KO mice (5 mice for mitochondrial respiratory capacity assay and 12 mice for mitochondrial swelling assay, respectively) were included in the study. All animals were housed in temperature-controlled rooms (24–28 °C) under a 12 h light-dark cycle (lights on at 7:00 AM, lights off at 7:00 PM) and had free access to food and water. Five mice were housed per cage. Environmental enrichment was provided. For the experiments, mice were anesthetized with 8% sevoflurane and then sacrificed by decapitation, and brain tissue samples were collected immediately. The allocation, the conduct of the experiment, the outcome assessment, and the data analysis were conducted with Dr. Kawachi aware of the group assignments.

### 2.2. Mitochondrial Respiratory Capacity Assay

**Figure 1:**
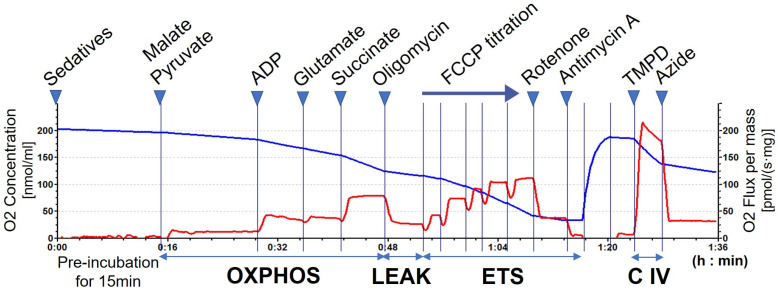
Representative SUIT protocol of mitochondrial respiratory measurement of homogenized mouse brain cortex. Oxygen concentration is shown by the blue line (left Y-axis) and oxygen flux is represented by the red line (right Y-axis) as a function of time. The “Oxygen flux” parameter represents the oxygen consumption rate. OXPHOS capacity was assessed by injection of pyruvate, malate, ADP, glutamate, and succinate, then LEAK respiration was assessed by injection of oligomycin. Then, ETS was assessed from the maximum oxygen consumption rate that reached a plateau in the FCCP titration. Finally, C IV activity was assessed by injection of TMPD and sodium azide, after injection of rotenone and antimycin A.

Mitochondrial respiration measurements were performed at 37 °C with stirring in 2 mL glass chambers using an Oroboros O2k (Oroboros Instruments, Innsbruck, Austria). All experiments were conducted using the MiR05 medium (sucrose 110 mM, HEPES 20 mM, taurine 20 mM, K-lactobionate 60 mM, MgCl_2_ 3 mM, KH_2_PO_4_ 10 mM, EGTA 0.5 mM, and 1 g/L bovine serum albumin, pH 7.1). Data were recorded using DatLab software (version 5.2.1.51, Oroboros Instruments). The instrument background and air calibrations were performed according to the manufacturer’s instructions. Cerebral cortex samples from 8–12-week-old male WT or CypD KO mice were homogenized using a Teflon pestle tissue grinder with a 9-fold excess of cold MiR05 on ice, and 20 μL of the homogenate (equivalent to 2 mg of tissue) was added to MiR05 containing various sedatives to adjust the total volume to 2 mL. As commonly used sedatives, dexmedetomidine hydrochloride (0.01, 0.1, 1, 10 μM; FUJIFILM Wako Pure Chemical Corporation, Osaka, Japan), midazolam (0.03, 0.3, 3, 30 μM; Maruishi Pharmaceutical Co., Ltd., Osaka, Japan), and propofol (50, 100, 150, 200 μM 2,6-diisopropylphenol; FUJIFILM Wako Pure Chemical Corporation) were evaluated in this study. These sedatives are kept under strict control, protected from light and stored in a lockable cabinet to maintain stability, and the condition of each anesthetic used is checked every time to prevent loss. The maximum concentrations of each were approximately 10 times the clinical range. In addition to the sedatives, calcium chloride (CaCl_2_; 500 or 1000 μM) was also used as positive control for inducing mPTP opening. The mixture was pre-incubated in chamber at 37 °C for 15 min before measurement. Then, substrates or inhibitors of mitochondrial respiratory system (5 mM malate, 5 mM pyruvate, 1 mM adenosine diphosphate (ADP), 5 mM glutamate, 20 mM succinate, 1 μg/mL oligomycin, 2 μM rotenone, 1 μg/mL antimycin A, 0.5 mM N.N.N′.N′-tetramethylphenylenediamine (TMPD), and 10 mM sodium azide) were multititrated (so called SUIT protocol, Substrate–Uncoupler–Inhibitor–Titration) as shown in Figure 1. Maximal uncoupled respiration was also induced after oligomycin addition by stepwise titrating the protonophore carbonyl cyanide-p-trifluoromethoxyphenyl-hydrazon (FCCP; 200 nM/addition) until there was no further increase in respiration. All chemicals were purchased from Sigma-Aldrich (St. Louis, MO, USA) unless otherwise stated. From the change in oxygen consumption rate, parameters such as oxidative phosphorylation (OXPHOS), proton leak (LEAK), electron transport system (ETS), and respiratory complex IV (C IV) activity of the test sample were calculated. Furthermore, the activities of respiratory complex I (C I) and II (C II) were calculated for propofol. C I activity was calculated by subtracting the respiration rate after rotenone addition from the maximum oxygen consumption rate determined by FCCP titration, and C II activity was calculated by subtracting the residual respiration rate after antimycin A addition from the respiration rate after rotenone addition. Each respiratory capacity values were normalized to the value for mitochondria from WT mice without the addition of sedatives. In addition, to examine whether the decrease in mitochondrial respiratory activity caused by propofol is reversible, the homogenate was incubated in the presence of 200 μM propofol at 37 °C for 1 h. And then 20 μL of the homogenate was diluted with 1980 μL of MiR05 containing 200 μM propofol or no propofol (final concentration 2 μM propofol), and similar measurements were performed. Propofol-untreated samples were used as controls. Data were obtained from five biological replicates.

### 2.3. Mitochondrial Swelling Assay

Isolation of brain mitochondria was performed according to the method of Hansson et al. [20,21,22]. In brief, approximately 100 mg of cerebral cortex samples from 8–12-week-old male WT or CypD KO mice were homogenized with 900 mg of cold isolation buffer (IB; 320 mM Sucrose, 2 mM EGTA, and 10 mM Trizma base, pH 7.4) containing 12% (*v*/*v*) Percoll solution, then, mitochondrial fractions were achieved using a discontinuous Percoll gradient of 26% and 40%, and diluted in IB to a fixed concentration after protein quantification according to Bradford [23] using bovine serum albumin as standard. Swelling experiments were performed according to the method of Morota et al. [24]. Briefly, the decrease in light scattering at 520 nm (reflecting mitochondrial swelling) of mitochondrial fraction (25 μg/mL) in the presence of dexmedetomidine hydrochloride (10 μM), midazolam (30 μM), or propofol (50, 100, 150, 200 μM) were measured with a fluorescence spectrometer LS-55 (PerkinElmer, Inc. Waltham, MA, USA) under de-energized conditions [22] at 28 °C using isotonic potassium chloride (KCl) buffer (containing 150 mM KCl, 20 mM 3-(N-morpholino) propanesulfonic acid, 10 mM Trizma base, 2 mM nitrilotriacetic acid, 0.5 μM rotenone, 0.5 μM antimycin A, and 2 μM Ca^2+^ ionophore (A23187)) at pH 7.3. The ionophore alamethicin (7.5 μg/mL) was added to induce a standardized maximum swelling response. A sample without sedatives was used as a control. The change when alamethicin was added to the control was taken as 100%, and the swelling ratio was calculated from the change in light scattering intensity when each sedative was added. Data were obtained from three biological replicates.

### 2.4. Detection of Cyt c by WB Analysis

The homogenate for mitochondrial respiration measurements (equivalent to 100 mg of tissue) was incubated with propofol (at concentrations of 150 or 200 µM) or CaCl_2_ (500 or 1000 µM) at 37 °C for 1 h, in addition, untreated homogenate was prepared as control. These homogenates were then separated into mitochondrial and cytosolic fractions by two-step centrifugation at low speed (1000× *g*, 5 min) and high speed (12,000× *g*, 5 min). In The mitochondrial fraction pellet was lysed in RIPA buffer (Santa Cruz Bioteconlogy, Santa Crus, CA, USA), containing 2 mM phenylmethylsulfonyl fluoride, 1 mM sodium orthovanadate, and protease inhibitor cocktail, kept on ice. The lysates were centrifuged at 10,000× *g* for 15 min at 4 °C. After protein quantification by Bradford method, the supernatants and cytosolic fractions were diluted in Laemmli sample buffer with 2-mercaptoethanol (Bio-Rad Laboratories, Hercules, CA, USA) and heated at 100 °C for 10 min. The samples containing 30 μg of protein per lane each were electrophoresed on a 12% precast gel (TGX, Bio-Rad Laboratories) at 100 V for 60 min and transferred by using Trans-Blot Turbo Transfer System (Bio-Rad Laboratories). After blocking, the membranes were incubated with antibodies in blocking solution. Anti-cytochrome c (136F3) rabbit monoclonal antibody (Cell Signaling Technology, Danvers, MA, USA, #4280S) or anti-GAPDH rabbit polyclonal antibody (Abcam, Cambridge, Cambridgeshire, UK, ab9485) were used as primary antibodies, and peroxidase-labeled anti-rabbit IgG(H + L) antibody (SeraCare Life Sciences, Milford, MA, USA, #5450-0010) was used as secondary antibody. After each antibody treatment, the membrane was washed three times in Tris-buffered saline with 0.05% Tween 20. The protein bands were detected with ECL Select Western blotting Detection Reagent (GE Healthcare Life Sciences, Chicago, IL, USA, #RPN2235). The luminescent images were acquired with the ChemiDoc XRS Plus System (Bio-Rad Laboratories). The resulting signal was quantified and background corrected using the software Image Lab (version 4.0.1, Bio-Rad Laboratories), and the cyt c/GAPDH ratio was calculated and evaluated. Data were obtained from three biological replicates.

### 2.5. Statistical Analysis

Statistical analysis was performed using Excel and the statistical software EZR (version 1.68) [25]. A comparison of three or more datasets was performed using a one-way analysis of variance (one-way ANOVA) followed by Dunnett’s test. Student’s *t*-test was used to compare the two groups. The data are expressed as means ± SD, and differences were considered statistically significant when the *p*-value was less than 0.05 (* *p* < 0.05, ** *p* < 0.01).

## 3. Results

### 3.1. Effects of Sedatives on Mitochondrial Respiratory Capacity

**Figure 2:**
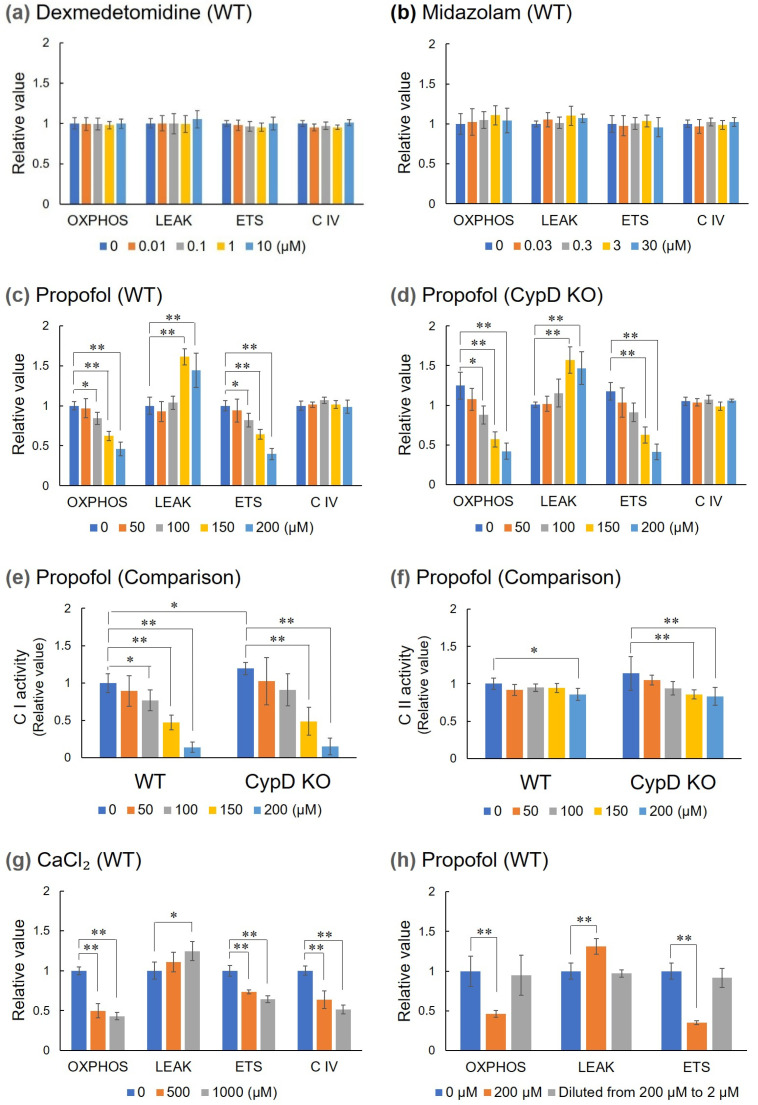
Effects of sedatives on mitochondrial respiratory capacity. Respiratory capacity parameters (OXPHOS, LEAK, ETS, and C IV) were calculated by measuring mitochondrial respiration in the presence of various sedatives. The Y-axis indicates the relative value of each respiratory capacity based on the value of WT without the addition of sedatives. All data are expressed as means ± SD (*n* = 5). Significance levels are indicated as follows: * *p* < 0.05, ** *p* < 0.01. (**a**–**c**) Changes in mitochondria from WT mouse brain upon addition of dexmedetomidine, midazolam, or propofol, respectively. (**d**) Changes in mitochondria from CypD KO mouse brain upon addition of propofol. Propofol also had a similar inhibitory effect on CypD KO. (**e**,**f**) Comparison of the effects of propofol on the activity of C I and II between WT and CypD KO. (**g**) Changes in mitochondria from WT mouse brain upon addition of CaCl_2_ to induce mPTP opening. In addition to the same decrease in respiratory capacity as with propofol addition, a decrease in C IV activity was also observed. (**h**) Reversibility of propofol-induced inhibition of mitochondrial respiration. The homogenate was incubated in the presence of 200 μM propofol for 1 h and then diluted 100-fold with MiR05 containing 200 μM propofol (orange column) or no propofol (gray column). Propofol-untreated samples were used as controls (blue column).

In the mitochondrial respiratory assay in WT mice, dexmedetomidine and midazolam had no effect in the range of concentrations tested (Figure 2a,b), but only propofol showed a dose-dependent decrease in OXPHOS and ETS, and increase in LEAK at concentrations above 150 µM (Figure 2c). Focusing on the effect of propofol, we compared it with the effect on mitochondria from CypD KO mice brain. Although CypD KO mice had higher mitochondrial OXPHOS and ETS values than WT mice, the addition of propofol abolished these advantages and caused respiratory depression similar to that in WT mice (Figure 2d). Furthermore, propofol-induced respiratory inhibition was more pronounced in CI than in CII for each respiratory chain complex, but CypDKO did not ameliorate this inhibition (Figure 2e,f). Addition of calcium instead of sedatives to induce mPTP opening caused not only a decrease in OXPHOS and ETS and an increase in LEAK, but also a significant decrease in the activity of respiratory C IV (Figure 2g), but this decrease in respiratory C IV activity was not observed with propofol (Figure 2c). In addition, when the homogenate was pretreated with 200 μM propofol for 1 h at 37 °C and then diluted to a final propofol concentration of 2 μM in MiR05, mitochondrial respiratory capacity was recovered to a level equivalent to that of the control (Figure 2h). Results of oxygen consumption rates measured by O2k are shown in Supplementary Materials (Figure S1).

### 3.2. Effects on Mitochondrial Swelling

**Figure 3:**
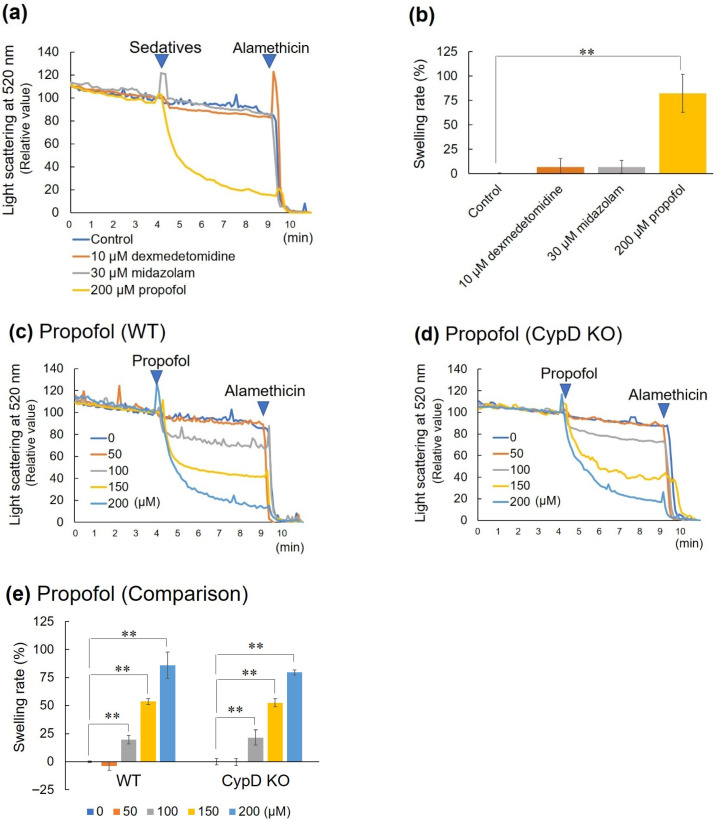
Effect on mitochondrial swelling. (**a**,**c**,**d**) Changes in light scattering intensity when various sedatives were added to mouse brain mitochondria. A sample without sedatives was used as a control. The Y-axis indicates the relative light scattering intensity at 520 nm, with the value immediately before the addition of the sedative set to 100 and the value after the addition of alamethicin set to 0. (**b**,**e**) Mitochondrial swelling ratio calculated from the change in light scattering intensity. The Y-axis indicates the relative swelling ratio, with the change in the control after the addition of alamethicin set to 100%. All data are expressed as mean ± SD (*n* = 3), and the significance levels are indicated as follows: ** *p* < 0.01. Panel (**a**) shows the change in light scattering intensity when various sedatives (10 μM dexmedetomidine, 30 μM midazolam, or 200 μM propofol) were added to WT mouse brain mitochondria, and panel (**b**) shows the swelling rate. Panels (**c**,**d**) show the change in light scattering intensity when propofol was added to WT or CypD KO mouse brain mitochondria, respectively, and panel (**e**) shows a comparison of the swelling rate.

The change in light scattering intensity when 10 μM dexmedetomidine, 30 μM midazolam, or 200 μM propofol (each the maximum concentration in this study) was added to WT mouse brain mitochondria showed that only propofol caused mitochondrial swelling (Figure 3a,b). To obtain more detailed data, various concentrations of propofol were added to WT mouse brain mitochondria, and a dose-dependent decrease in light scattering intensity was observed at concentrations of 100 μM or more (Figure 3c). Similar results were obtained in CypD KO mouse brain mitochondria (Figure 3d). The change in mitochondrial swelling ratio calculated from these results was almost the same in both cases, and propofol-induced mitochondrial swelling was not affected by CypD KO (Figure 3e).

### 3.3. Detection of Cyt c by WB

**Figure 4:**
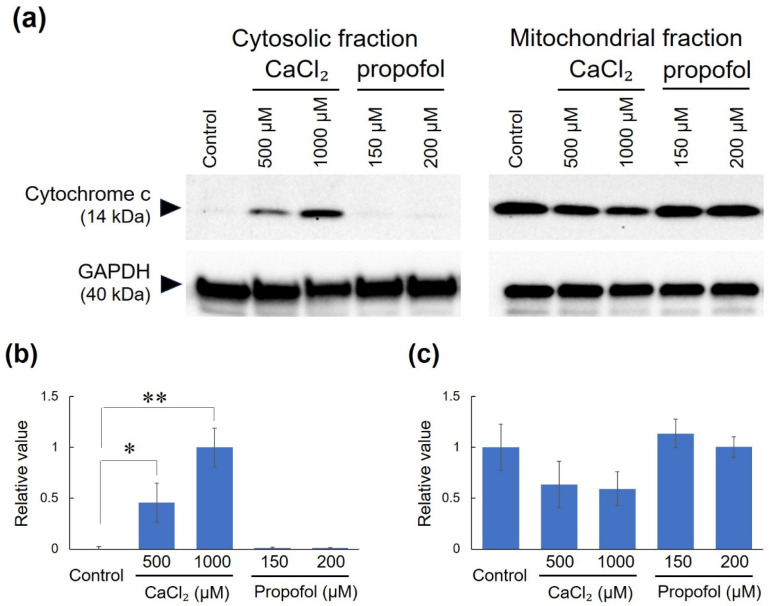
Detection of cyt c by WB. (**a**) Cyt c signal in the cytosolic or mitochondrial fraction of mouse brain homogenate exposed to high concentrations of calcium chloride or propofol for one hour. GAPDH signal was also detected as an internal control. (**b**,**c**) Quantified cyt c/GAPDH ratio in the cytosolic and mitochondrial fractions, respectively. The Y-axis indicates relative values, relative to the maximum value in (**b**) and to the control value in (**c**). All data are expressed as mean ± SD (*n* = 3), and the significance levels are indicated as follows: * *p* < 0.05, ** *p* < 0.01.

After exposure to high concentrations of propofol or calcium chloride, WT mouse brain homogenates were separated into mitochondrial and cytosolic fractions by centrifugation, and cyt c in each protein solution was detected by WB. The calcium-treated samples showed a strong cyt c signal in the cytosolic fraction, with a correspondingly reduced signal in the mitochondrial fraction. In contrast, the propofol-treated samples showed almost no cyt c signal in the cytosolic fraction (Figure 4a). Normalization of the quantified cyt c signal to GAPDH levels clearly demonstrated release of cyt c from mitochondria to the cytosol in the calcium-treated samples, whereas propofol-treated samples showed no cyt c release, similar to that observed in the untreated samples (Figure 4b,c). Original blots of all replicates used for WB statistical analysis are shown in Supplementary Materials (Figure S2).

## 4. Discussion

In this study, we investigated the effects of sedative drugs on mitochondrial function in mouse brains, with a particular focus on propofol, which was found to influence mitochondrial respiratory capacities. To examine the role of mitochondrial permeability transition in propofol-induced toxicity, we compared wild-type mice to CypD KO mice and analyzed the potential swelling-induced release of cyt c. Mitochondrial dysfunction, which disrupts cellular homeostasis, can lead to apoptosis via reactive oxygen species (ROS)-mediated pathways [26]. Specifically, ROS are mainly generated by complexes I and III in the electron transport chain, and, similar to calcium, contribute to the opening of the mPTP, thereby activating the mitochondrial apoptotic pathway [27,28,29]. This process is followed by the release of cyt c into the cytosol, where it activates caspase enzymes. In our study, propofol inhibited mitochondrial respiration by directly impairing the ETS. And the inhibition of ETS was mainly due to a decrease in C I activity, which is consistent with previous reports that C I is the main target of propofol toxicity [30,31]. The propofol concentrations we used, 50–200 μM, are higher than the reported clinical range of 11–27.5 μM for plasma concentrations during anesthesia [32]. However, studies in rats have demonstrated that tissue concentrations of propofol can reach 200 μM following the administration of 20 mg/kg under certain conditions [31], and high concentrations of propofol are known to induce cell death [11,33]. This high concentration situation may also occur in PRIS, which is thought to be caused by prolonged exposure to high doses of propofol. At “toxic” concentrations (100–200 μM) above the clinical range, propofol inhibited OXPHOS and ETS, while simultaneously increasing LEAK, which suggests a reduction in ATP production and a potential increase in ROS generation. The propofol-induced suppression of OXPHOS could trigger a metabolic shift from oxidative phosphorylation to glycolysis, potentially enhancing lactate production [11,34,35]. This condition may mimic brain ischemia [36].

The effects of propofol on mitochondrial respiratory capacity resemble those induced by calcium, with the exception of the cyt c oxidase (C IV) response. Cyt c plays a critical role in mediating apoptosis [37,38,39] and is a key component of the ETS, facilitating electron transfer between complexes III and IV [40]. While calcium inhibits C IV activity, propofol did not exhibit this effect. Since TMPD, an activator of cyt c, is used as a substrate for C IV, our findings suggest that cyt c remains within the mitochondria even after propofol administration, in contrast to the effects of calcium. Furthermore, the propofol-induced mitochondrial respiratory dysfunction was reversible upon reducing propofol concentration, supporting the notion that this impairment is not permanent.

A previous in vivo study has indicated that the dose of propofol required to induce neuronal apoptosis (in the infant mouse brain) is approximately one-quarter of that needed for surgical anesthesia [9,41]. Mitochondrial dysfunction induced by propofol has been shown to include reductions in mitochondrial mass, the mitochondrial-to-nuclear DNA ratio, intracellular ATP production, mitochondrial respiratory rate, and cyt c oxidase activity, alongside increased mitochondrial ROS production [9]. It is well established that calcium addition to mitochondria promotes mPTP opening, mitochondrial swelling, and cyt c release into the cytosol, effects that can be inhibited by CypD inhibitors [42]. In contrast, propofol-induced mitochondrial swelling occurred in a dose-dependent manner, with similar results observed in mitochondria from both wild-type and CypD KO mice, suggesting that mPTP opening, as mediated by CypD, does not contribute to propofol-induced mitochondrial swelling. This was further supported by WB analysis, which did not reveal cyt c release into the cytosol following propofol treatment.

Taken together, our results suggest that propofol induces mitochondrial dysfunction, including reduced respiratory capacity and swelling, but these effects appear to be independent of mPTP opening. The mPTP is a crucial mediator of apoptosis and necrosis, and its opening can be specifically blocked by cyclosporin A (CsA), a CypD inhibitor [22,43]. CypD is an essential component of the mPTP, and its knockout is known to inhibit mPTP opening and stabilize mitochondrial function [17,44,45]. However, our data indicate that propofol-induced mitochondrial dysfunction and swelling were unaffected by CypD deletion, and no cyt c release into the cytosol was observed. These findings also align with the observation that C IV activity, when measured using TMPD as a substrate, was not inhibited by propofol. We have previously evaluated the effect of propofol on calcium-induced mitochondrial swelling and calcium retention capacity [24]. To our knowledge, this represents the first report of propofol’s direct effects on mouse brain mitochondrial swelling.

Our results suggest that propofol has a “toxic” effect on mitochondria, causing respiratory dysfunction and swelling. However, the precise mechanisms underlying these effects remain unclear. We propose that the observed respiratory dysfunction and swelling may arise from detergent-like perturbations of membrane integrity, excessive production of ROS, or disruption of mitochondrial exchangers. However, although these mechanisms are plausible and mechanistically consistent with the swelling and respiration defects, they remain experimentally untested. Accordingly, we now plan follow-up experiments—including Amplex Red-based ROS quantification, TMRM assays for mitochondrial membrane potential (ΔΨm), and Ca^2+^-retention capacity measurements—as the foundation that motivates subsequent investigations in cellular models. Such studies will be required to fully delineate the pathways and mechanisms through which propofol elicits these effects.

This study has several limitations. First, the experimental model used in this study was in vitro and focused on the direct effects of propofol on mitochondria in brain tissue. Thus, the complex in vivo interactions of propofol with other cellular components were not considered. We cannot exclude the involvement of various factors, such as Bax, Bad, Bcl-2, TNF, caspases, ROS, and others, in the induction of mPTP [46,47,48,49]. In this study, 2,6-diisopropylphenol was used as the experimental reagent, but it should also be noted that clinical propofol is a lipid emulsion. Furthermore, although BSA has been reported to bind to propofol and inhibit its function [50], this study did not consider the effect of BSA in MiR05 on the interpretation of propofol dosage. The de-energized swelling test has the limitation that it does not necessarily correspond to physiological conditions, and propofol-induced mitochondrial swelling could also be influenced by the activation of the GABA_A_ receptor and intracellular calcium levels [51]. Furthermore, excessive propofol concentrations (200 μM) has been shown to impair autophagic flux and function through overactivation of InsP3R and RYR channels, promoting neuronal injury [52]. In the present study, pharmacological mPTP inhibition using CsA to counteract propofol-induced mitochondrial swelling was preliminary tested, but was excluded from this report due to insufficient number of replicates, and will be confirmed in future studies. Thus, further research, including cellular models, is needed to fully elucidate the pathways and mechanisms by which propofol exerts these effects.

## Data Availability

The original contributions presented in this study are included in the article. Further inquiries can be directed to the corresponding author.

## Supplementary Materials

The following supporting information can be downloaded at: https://www.mdpi.com/article/10.3390/biomedicines13123125/s1, Figure S1: Results of oxygen consumption rates measured by O2k (corresponding to Figure 2), and Figure S2: Original blots of all replicates used for Western blot statistical analysis.

## Abbreviations

The following abbreviations are used in this manuscript:

ADP	adenosine diphosphate
ATP	adenosine triphosphate
CaCl_2_	calcium chloride
C I	complex I
C II	complex II
C IV	complex IV
CsA	cyclosporin A
CypD	cyclophilin D
cyt c	cytochrome c
ETS	electron transport system
FCCP	carbonyl cyanide-p-trifluoromethoxyphenyl-hydrazon
IB	isolation buffer
KCl	potassium chloride
KO	knockout
mPTP	mitochondrial permeability transition pore
OXPHOS	oxidative phosphorylation
PRIS	propofol infusion syndrome
ROS	reactive oxygen species
SUIT	Substrate–Uncoupler–Inhibitor–Titration
WB	Western blot
