# Supplementary figures and images for: Propofol-Induced Mitochondrial Dysfunction Is Independent of Mitochondrial Permeability Transition
Source: Biomedicines. 2025 Dec 18;13(12):3125. doi: 10.3390/biomedicines13123125 (PMC12730529; doi:10.3390/biomedicines13123125)

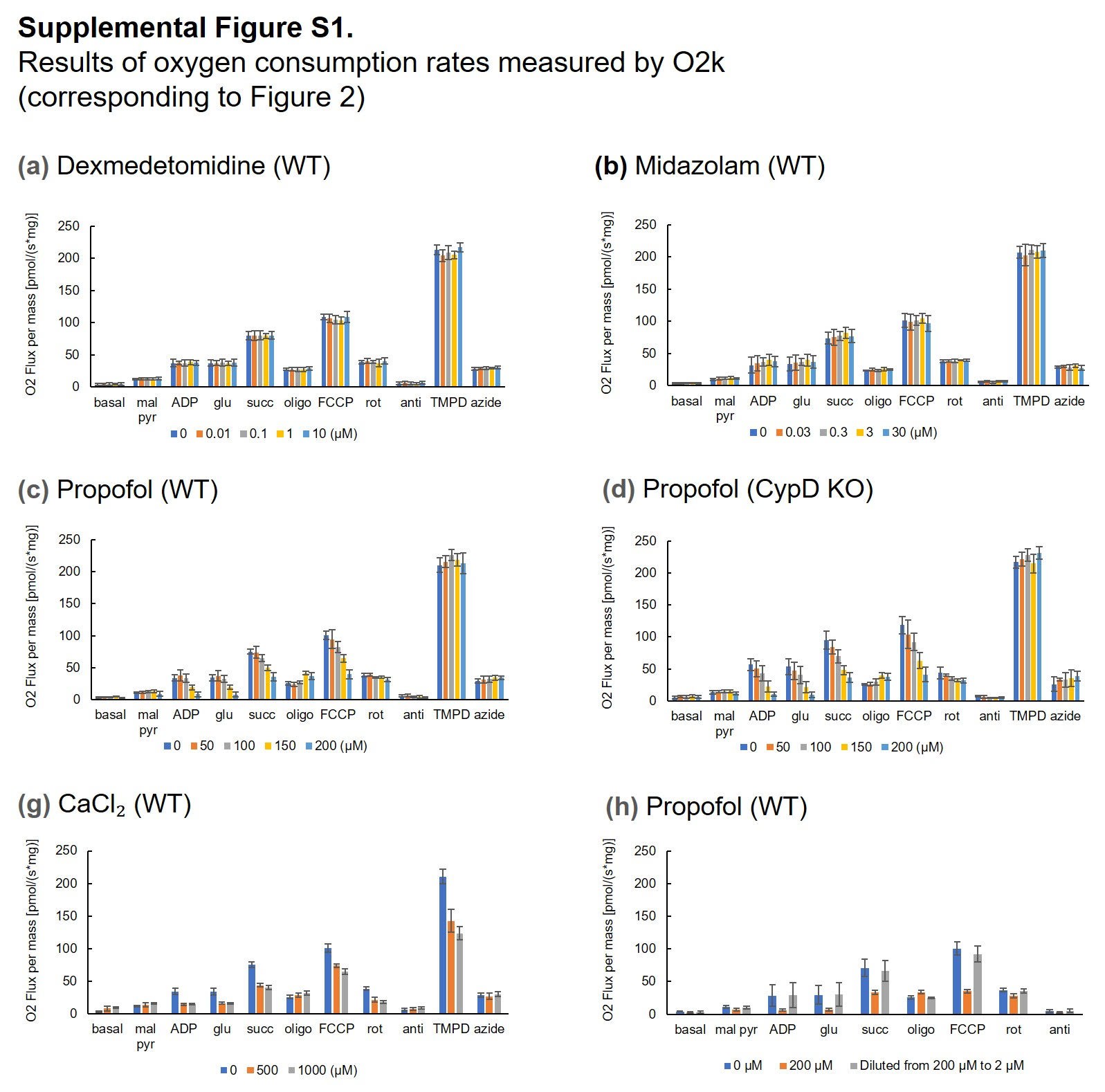

Supplement: Supplementary file 1 [file biomedicines-13-03125-s001.zip › Supplemental Figure S1.jpg]

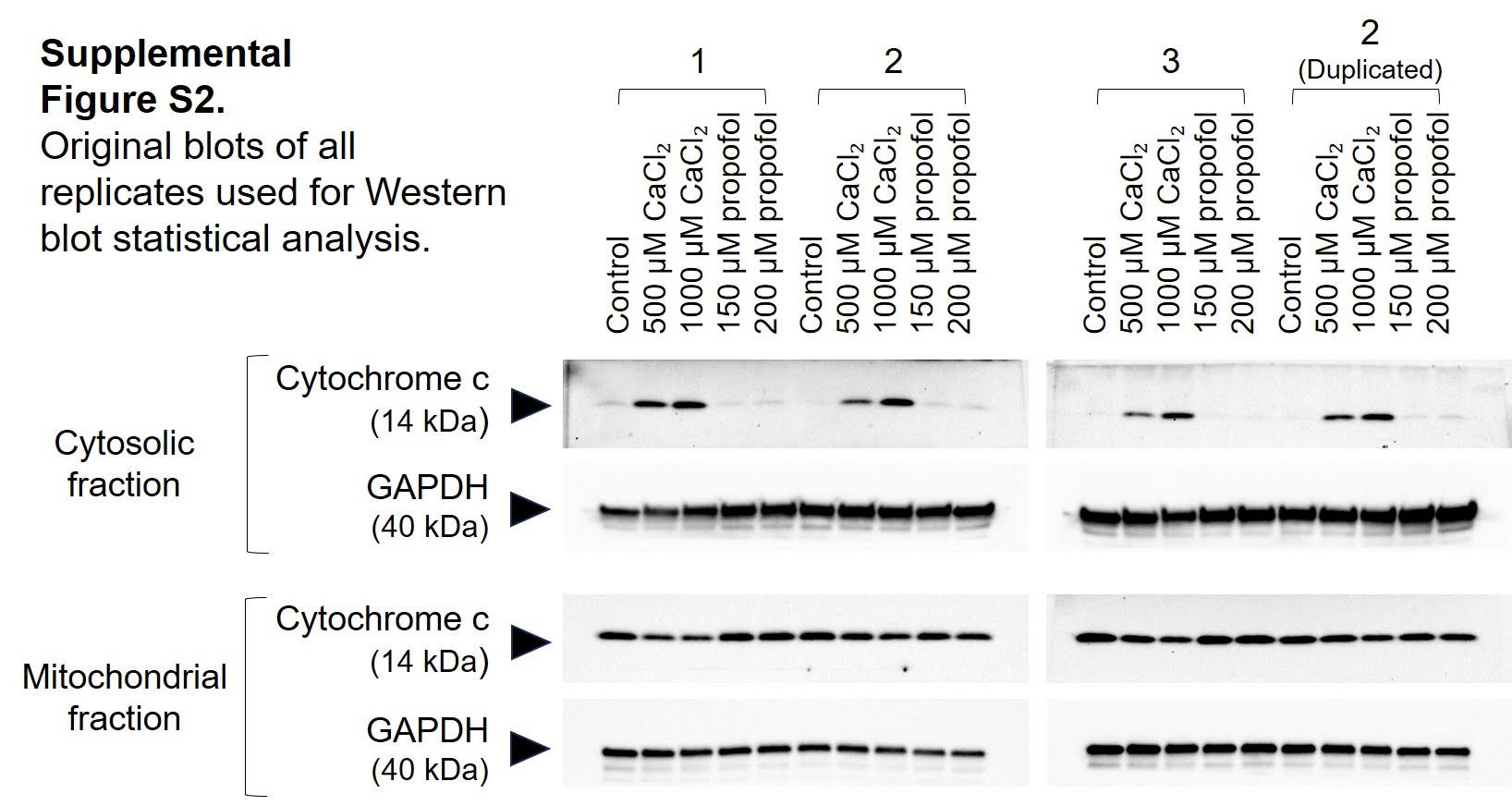

Supplement: Supplementary file 1 [file biomedicines-13-03125-s001.zip › Supplemental Figure S2.jpg]
